# Evaluation of the RSNA and CORADS classifications for COVID-19 on chest computed tomography in the Brazilian population

**DOI:** 10.6061/clinics/2021/e2476

**Published:** 2021-03-19

**Authors:** Eduardo Kaiser Ururahy Nunes Fonseca, Bruna Melo Coelho Loureiro, Daniel Giunchetti Strabelli, Lucas de Pádua Gomes de Farias, José Vitor Rassi Garcia, Victor Arcanjo Almeida Gama, Lorena Carneiro Ferreira, Rodrigo Caruso Chate, Antonildes Nascimento Assunção, Marcio Valente Yamada Sawamura, Cesar Higa Nomura

**Affiliations:** Hospital das Clinicas HCFMUSP, Faculdade de Medicina, Universidade de Sao Paulo, Sao Paulo, SP, BR.

**Keywords:** COVID-19, Viral, Pneumonia, Tomography, X-Ray Computed, Pandemics

## Abstract

**OBJECTIVE:**

To determine the correlation between the two tomographic classifications for coronavirus disease (COVID-19), COVID-19 Reporting and Data System (CORADS) and Radiological Society of North America Expert Consensus Statement on Reporting Chest Computed Tomography (CT) Findings Related to COVID-19 (RSNA), in the Brazilian population and to assess the agreement between reviewers with different experience levels.

**METHODS:**

Chest CT images of patients with reverse transcriptase-polymerase chain reaction (RT-PCR)-positive COVID-19 were categorized according to the CORADS and RSNA classifications by radiologists with different levels of experience and who were initially unaware of the RT-PCR results. The inter- and intra-observer concordances for each of the classifications were calculated, as were the concordances between classifications.

**RESULTS:**

A total of 100 patients were included in this study. The RSNA classification showed an almost perfect inter-observer agreement between reviewers with similar experience levels, with a kappa coefficient of 0.892 (95% confidence interval [CI], 0.788-0.995). CORADS showed substantial agreement among reviewers with similar experience levels, with a kappa coefficient of 0.642 (95% CI, 0.491-0.793). There was inter-observer variation when comparing less experienced reviewers with more experienced reviewers, with the highest kappa coefficient of 0.396 (95% CI, 0.255-0.588). There was a significant correlation between both classifications, with a Kendall coefficient of 0.899 (*p*<0.001) and substantial intra-observer agreement for both classifications.

**CONCLUSION:**

The RSNA and CORADS classifications showed excellent inter-observer agreement for reviewers with the same level of experience, although the agreement between less experience reviewers and the reviewer with the most experience was only reasonable. Combined analysis of both classifications with the first RT-PCR results did not reveal any false-negative results for detecting COVID-19 in patients.

## INTRODUCTION

Initial studies evaluating chest computed tomography (CT) findings in patients with coronavirus disease (COVID-19) showed a high sensitivity of 94% but a low specificity of 37% (1). However, during the COVID-19 pandemic two classifications for chest CT findings were developed (2,3) - COVID-19 Reporting and Data System (CORADS) and Radiological Society of North America Expert Consensus Statement on Reporting Chest CT Findings Related to COVID-19 (RSNA) - which have a high sensitivity and high specificity, even in the Brazilian population (4-9). In addition, chest CT has proven useful in the initial evaluation of patients, not only because of its rapid results, as it detects changes suggestive of infection in minutes in contrast to reverse transcriptase-polymerase chain reaction (RT-PCR) and serological tests, which can sometimes take days, but also because of its capacity to assess the initial extent of disease, information that can be used as a tool in initial decision-making and that is correlated with prognosis (10,11).

**Figure 1:**
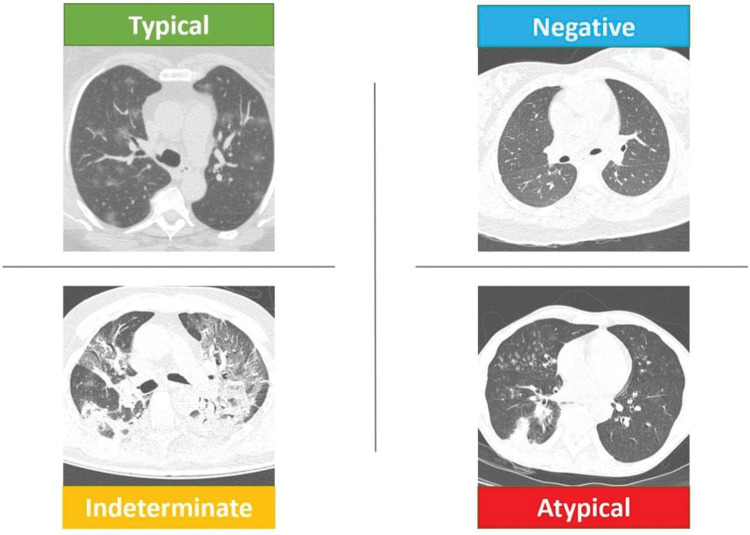
Schematic representation of the RSNA classification. The upper left image shows “typical” CT findings of COVID-19 with multifocal rounded ground-glass opacities. The lower left image shows an “indeterminate” CT findings of COVID-19 with perihilar bilateral opacities and a lack of “typical” tomographic findings. The lower right image shows “atypical” CT findings of COVID-19 with lower lobe consolidation and bilateral “tree-in-bud” images. The upper right image shows “negative” CT findings without signs of infection. COVID-19, Coronavirus disease; CT, computed tomography; RSNA, Radiological Society of North America.

The consensus of experts from the North American Radiological Society, endorsed by the Thoracic Radiology Society and the American College of Radiology (RSNA consensus) (2), was published in March 2020 to help radiologists recognize COVID-19 pneumonia on chest CT. According to the authors, the purpose of this consensus was to help radiologists communicate with other health professionals, thus allowing faster management of COVID-19 patients. In this consensus, four categories were proposed to report CT findings potentially attributable to COVID-19, on the basis of CT findings reported in the literature and their typicality in COVID-19 pneumonia rather than other diseases. The categories were: 1) typical appearance (bilateral peripheral ground-glass opacities, multifocal ground-glass opacities with rounded morphology, reversed halo sign, or other organizing pneumonia findings); 2) indeterminate appearance (multifocal, diffuse, peri-hilar, or unilateral ground-glass opacities, without peripheral distribution and not rounded or few and very small ground-glass opacities); 3) atypical appearance (isolated lobar or segmental consolidation without ground-glass opacities, discrete small nodules (centrilobular, “tree-in-bud”), pulmonary cavitation, smooth interlobular septal thickening with pleural effusion; and 4) negative for pneumonia. As previously mentioned, this classification leads better specificity in tomographic findings. Figure 1 exemplifies this classification.

**Figure 2:**
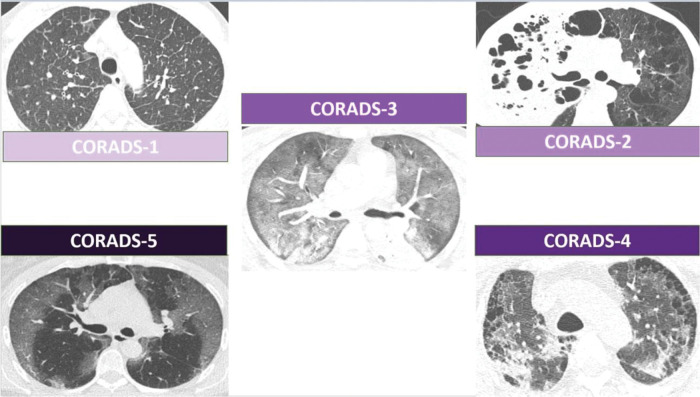
Schematic representation of the CORADS classification. The upper left image shows CORADS-1 CT findings without signs of infection but shows interlobular septa thickening, suggesting lung congestion. The upper right image shows CORADS-2 CT findings, with extensive consolidation of the right upper lobe with areas of cavitation. There is also widespread lung emphysema. The central image shows CORADS-3 CT findings, with diffuse ground-glass opacities. The lower right image shows CORADS-4 CT findings, with peripheral bilateral ground-glass opacities in a patient with emphysema. The lower left image shows CORADS-5 CT findings, with peripheral bilateral ground-glass opacities. CORADS, COVID-19 reporting and data system; CT, computed tomography.

In April 2020, the COVID-19 Reporting and Data System (CORADS) (3) was published for a standardized assessment of COVID-19 pulmonary involvement in chest CT findings. Developed by the Dutch Radiological Society, CORADS assesses the suspected pulmonary involvement of COVID-19 and classifies patients on the following scale: 0, not interpretable; 1, very low probability; 2, low probability; 3, uncertain; 4, high probability; 5, very high probability; and 6, confirmed. Figure 2 exemplifies this classification.

According to the authors, the CORADS system showed moderate-to-substantial agreement between observers, with a total Fleiss’ kappa of 0.47 (95% confidence interval [CI], 0.45-0.49) and high discriminatory power to diagnose COVID-19, with a mean area under the receiver operating characteristic curve of 0.91 (95% CI, 0.85-0.97) for positive results from RT-PCR.

However, few studies have addressed the application of these classifications in the Brazilian population, and there are no studies comparing them in this population. In addition, few studies have evaluated the impact of previous experience with chest radiology on the use of this type of classification, comparing users with different levels of experience.

Therefore, the objective of this study was to compare the application of the two existing tomographic classifications for COVID-19 (CORADS and RSNA consensus) in the Brazilian population by radiologists with different levels of experience.

## MATERIALS AND METHODS

### Study design and population

This observational and retrospective study was approved by our institutional review board, and the requirement to obtain written informed consent was waived. The procedures were performed in accordance with guidelines in the Helsinki Declaration.

A total of 100 patients with a final diagnosis of COVID-19 who had undergone chest CT were randomly selected from a group of 1278 patients hospitalized in a tertiary hospital in Brazil (Complex of the Hospital das Clínicas, Faculty of Medicine, University of São Paulo) between 03/16/2020 and 05/13/2020.

Exclusion criteria were as follows: (a) patients who had not been tested or did not have a positive final RT-PCR result for severe acute respiratory syndrome coronavirus-2 (SARS-CoV-2); (b) patients who did not undergo chest CT; and (c) the presence of an important motion artifact on chest CT.

### Clinical, laboratory, and tomographic data

Analysis of the electronic medical records of all selected patients was performed with a search for clinical characteristics (age, sex, comorbidities, symptoms) and RT-PCR results (collected in up to three samples, when the clinical suspicion remained in patients with negative results).

All included patients had undergone chest CT according to the following protocol: supine position during maximum inspiration, without intravenous contrast; in a scanner with at least 64 channels; using collimation, voltage (kV), and tube current (mAs) according to institutional protocols already established for each device and patient biotype. The tomographic images were accessed through an integrated picture archiving and communication system.

### Analysis of tomographic images

The CT findings were evaluated by radiologists with 1, 2, and 7 years of experience in chest imaging. Each reviewer with 1 and 2 years of experience evaluated the images using CORADS or RSNA, and the most experienced reviewer evaluated each case at two different times, with a 1-week interval, and in random order, applying both classifications. The evaluators did not have access to the results of RT-PCR until the end of the analyses. All patients were categorized on the basis of the RSNA consensus classification (typical, indeterminate, atypical, or negative for pneumonia) and CORADS (1=very low probability; 2=low probability; 3=uncertain; 4=high probability; 5=very high probability). The categories CORADS categories 0=not interpretable and 6=confirmed were not applicable.

Altogether 20% of chest CT images were randomly selected and reclassified by the most experienced evaluator three months after the first analysis, allowing for the calculation of intra-observer agreement.

### Statistical analyses

Statistical analyses were performed using SPSS for Windows (Version 25, Armonk, USA: IBM Corp.). Data are presented as mean±standard deviation or median and interquartile range on the basis of their normality. To quantify the inter-observer and intra-observer agreement, Fleiss’ kappa was calculated, reported as absolute values and 95% CIs. Inter-observer agreement was considered weak for kappa values of 0.01-0.20, reasonable for kappa values of 0.21-0.40, moderate for kappa values of 0.41-0.60, substantial for kappa values of 0.61-0.80, and almost perfect for kappa values of 0.81-1.00. Considering the ordinal categorical characteristics of the variables under analysis, the CORADS and RSNA classifications were correlated using Kendall’s tau-b correlation coefficient. For the analysis of agreement between classifications, categories CORADS-1 and -2 were grouped and considered equivalent to atypical RSNA, and category CORADS-3 was considered equivalent to the indeterminate category. CORADS-4 and -5 were grouped and considered equivalent to the typical RSNA category, according to the original CORADS article (6).

## RESULTS

One hundred patients were included in the study. The mean age of the patients was 54±18 years (range, 6 months to 93 years), with 52 (52%) being female. Table 1 summarizes the patients’ main demographic and clinical characteristics.

Of these 100 patients, 85 showed positive RT-PCR results after the first test; nine, after the second test; and six, after the third test. This resulted in a sensitivity of 85% (95% CI, 77-90%) for the RT-PCR test, with consecutive increments for subsequent samples.

### Categorization of patients by classification

Table 2 shows the distribution of the 100 patients according to the categorization by each of the reviewers for the RSNA and CORADS classifications.

### Inter-observer and intra-observer variability and agreement in classifications by the reviewers

The assessment of inter-observer agreement is shown in Table 3. The intra-observer analysis was based on the most experienced reviewer who showed substantial agreement for both classifications, with a kappa of 0.712 (95% CI, 0.430-0.993) for the RSNA consensus and 0.685 (95% CI, 0.426-0.944) for CORADS. Agreement analysis between classifications was also performed on the basis of the analysis of evaluator 3, which revealed an almost perfect agreement, with a kappa of 0.820 (95% CI, 0.714-0.926). Table 4 shows the agreement between classifications. The Kendall tau-b correlation coefficient between the two classifications was 0.899 (*p*<0.001).

## DISCUSSION

This study was developed with the primary intention of evaluating two tomographic classifications available for COVID-19 (RSNA consensus and CORADS) in the Brazilian population in the context of the current pandemic.

Our results showed that reviewers with similar chest CT experience tend to use both classifications in a similar manner, presenting substantial inter-observer (CORADS) and almost perfect (RSNA) agreements. However, this agreement index was lower when less experienced reviewers were compared with the more experienced reviewer, with a reasonable general agreement for both classifications, although with higher kappa values in the evaluation of the RSNA classification. This discrepancy is because of the greater number of cases classified as RSNA indeterminate and CORADS-3 by the most experienced reviewer.

The RSNA indeterminate/CORADS-3 classification generally reflects either very extensive disease in which the characteristic peripheral distribution pattern or rounded opacities are lost or initial disease in which there are few opacities so that it fits into a pattern more suggestive of COVID-19 pulmonary involvement (12-14). It is interesting to note that this category can be interpreted as either suggestive of pulmonary involvement by COVID-19 or not according to some recent articles (6,8,9) and that this impacts the sensitivity and specificity attributed to chest tomography. When considering the RSNA indeterminate category/CORADS-3 as “positive,” sensitivity increases substantially at the expense of specificity. The opposite occurs when this category is considered “negative.”

Drawing a parallel in our study, the most experienced reviewer was more specific and less sensitive in his classification, pointing out more cases as indeterminate and less as typical; the opposite occurred with less experienced reviewers.

When we consider only the atypical cases and CORADS-1 and -2, we note that there was no great divergence between reviewers with different levels of experience, despite the low number of cases in these categories. This shows that neither classification has a large number of false negatives (less than 10% of cases regardless of the years of experience - a rate lower than that calculated for the first RT-PCR in our study, with rates of 15%). This demonstrates the potential for using these classifications for excluding pulmonary involvement in COVID-19.

Furthermore, we highlight the complementary potential of CT with RT-PCR. In our sample, none of the cases whose first RT-PCR was negative were classified as atypical or CORADS-1 or -2 by the most experienced reviewer, and all cases that this reviewer considered atypical or CORADS-1 or -2 tested positive on the first RT-PCR; thus, when combining both tests none of the 100 COVID-19 patients in our study would have been considered false-negative cases.

We have highlighted substantial agreement between these classifications in our Brazilian population, as observed in other populations (3,7,9,15), and the consistency of the classification chosen by reviewer 3 who used the categories indeterminate and CORADS-3 more often than did other reviewers. It is also worth mentioning that the divergent cases (n=10) were classified as typical by RSNA and CORADS-3, without any disagreement in atypical cases and CORADS-1 and -2.

We also highlight the substantial intra-observer agreement found for both classifications even 3 months after the initial case analysis and in the absence of training, which at least in part may be because of the reviewer’s permanent and daily exposure to chest CT findings of COVID-19 patients.

Our study has some limitations. It was performed retrospectively and in a tertiary hospital, which may have led to selection biases, and our setting may limit the extrapolation of results to other healthcare environments with less severely ill patients. In addition, there was no training or calibration between the reviewers use of the classifications since the beginning of the pandemic. We only included patients with a diagnosis confirmed by RT-PCR, so it was not possible to extrapolate these results to patients with other diagnoses to calculate sensitivity and specificity; however, this has already been done in our country by other groups (4,5).

## CONCLUSION

This study revealed that reviewers with similar levels of experience showed substantial agreement for both classifications, although this agreement was only reasonable when these reviewers were compared with another more experienced one and was at the expense of a greater number of cases considered as RSNA “indeterminate”/CORADS-3.

There was no significant divergence of negative cases among the reviewers, regardless of the level of experience. When evaluated together with the first RT-PCR, there were no false-negative results for either classification in our patients.

These data corroborate the use of these tomographic classifications to aid in the diagnosis and management of patients with suspected COVID-19 in Brazil.

## AUTHOR CONTRIBUTIONS

All authors conceived and designed the study. Fonseca EKUN, Strabelli DG, Farias LPG, Ferreira LC, Garcia JVR, Gama VAA and Sawamura MVY collected the data. Fonseca EKUN, Loureiro BMC, Assunção Júnior AN, Chate RC, Sawamura MVY and Nomura CH analyzed the data. All authors helped writing the manuscript. All authors contributed to manuscript revisions, have approved the final version, and agree to be held accountable for the content therein.

## Figures and Tables

**Table 1:** Patient characteristics.

Age (mean±SD, years)	54±18
Female	52 (52%)
Comorbidities	
-Hypertension	57 (57%)
-Diabetes	27 (27%)
-Obesity	18 (18%)
-Cardiopathy	15 (14%)
-Asthma	3 (3%)
-COPD	11 (1%)
No comorbidity	15 (15%)
Symptoms duration (mean±SD, days)	7±5
Symptoms	
-Fever	62 (62%)
-Dyspnea	73 (73%)
-Cough	73 (73%)
-Sore throat	5 (5%)
-Headache	14 (14%)
-Muscle tenderness	35 (35%)

SD, Standard deviation; COPD, Chronic obstructive pulmonary disease.

**Table 2:** Categorization of patients by RSNA consensus and CORADS.

RSNA	Reviewer 1	Reviewer 2	Reviewer 3
Atypical	4 (4%)	3 (3%)	6 (6%)
Indeterminate	26 (26%)	19 (19%)	47 (47%)
Typical	70 (70%)	78 (78%)	47 (47%)

CORADS	Reviewer 1	Reviewer 2	Reviewer 3
CORADS-1	2 (2%)	2 (2%)	2 (2%)
CORADS-2	5 (5%)	3 (3%)	4 (4%)
CORADS-3	9 (9%)	6 (6%)	37 (37%)
CORADS-4	7 (7%)	16 (16%)	16 (16%)
CORADS-5	77 (77%)	73 (73%)	41 (41%)

CORADS, COVID-19 reporting and data system.

**Table 3:** Inter-observer agreement on the RSNA consensus and CORADS classifications between pairs of reviewers.

	kappa (95% CI)
	RSNA
Reviewer 1 *versus* 2	0.892 (0.788-0.995)
Reviewer 1 *versus* 3	0.396 (0.255-0.588)
Reviewer 2 *versus* 3	0.359 (0.220-0.537)

	CORADS
Reviewer 1 *versus* 2	0.642 (0.491-0.793)
Reviewer 1 *versus* 3	0.207 (0.091-0.323)
Reviewer 2 *versus* 3	0.226 (0.110-0.342)

Abbreviation: CI, confidence interval.

**Table 4:** Agreement between classifications.

RSNA	CORADS-1 and 2	CORADS-3	CORADS-4 and 5
Atypical	6	0	0
Indeterminate	0	37	10
Typical	0	0	47

CORADS, COVID-19 Reporting and Data System.
